# Lumpectomy May Negatively Impact Survival in Female Dogs with Mammary Carcinomas

**DOI:** 10.3390/vetsci12070631

**Published:** 2025-07-02

**Authors:** Sheila Santana de Mello, Aracelle Alves de Avila Fagundes, Francisco C. D. Mota, Alessandra A. M. Ronchi

**Affiliations:** 1 Graduate Program in Veterinary Medicine, Federal University of Uberlândia, BR-050, KM 78, Glória Campus, Block 1CCG, Uberlândia 38410-337, MG, Brazil; 2 Companion Animal Surgical Clinic Division, Veterinary Hospital, Federal University of Uberlândia, 3289 Mato Grosso Avenue, Umuarama Campus, Uberlândia 38405-314, MG, Brazil; aracelle.alves@ufu.br (A.A.d.A.F.); francisco.mota@ufu.br (F.C.D.M.); 3 Animal Pathology Laboratory, Veterinary Hospital, Federal University of Uberlândia, 3289 Mato Grosso Avenue, Umuarama Campus, Uberlândia 38405-314, MG, Brazil; alessandra.medeiros@ufu.br

**Keywords:** dog, mammary neoplasm, survival analysis, mastectomy

## Abstract

Mammary tumors are the most common neoplasms in female dogs, with mastectomy being the primary treatment. The impact of mastectomy type on survival remains unclear. This study evaluated the effect of different surgical techniques on the survival of female dogs with mammary carcinomas. A total of 307 dogs with 734 mammary tumors were analyzed. Ages ranged from 1 to 17 years, with mixed breeds comprising 48.9%. Most tumors (92.9%) were malignant, predominantly carcinoma in a mixed tumor (30.2%). Unilateral mastectomy was the most common technique (65.8%). Lumpectomy showed the shortest mean survival (179 days) and was associated with a 4.9-fold increased risk of death (*p* = 0.009). There was no significant association between surgical technique and overall survival or recurrence of mammary carcinoma (*p* = 0.2). This study relied on owner-reported data for survival and tumor recurrence. Lumpectomy appears to be associated with shorter survival times in female dogs with mammary carcinoma. Therefore, it may be advisable to reconsider this technique as a primary treatment option.

## Simple Summary

The impact of mammary carcinomas on the health and survival of dogs is a critical concern in veterinary oncology. Understanding the influence of surgical techniques on patient outcomes is essential for improving treatment strategies. This retrospective study analyzed the survival of female dogs with mammary carcinomas, comparing different mastectomy techniques over a 10-year period. We observed that lumpectomy was associated with a shorter survival time, highlighting the importance of selecting appropriate surgical approaches.

## 1. Introduction

Mammary tumors are the most frequent neoplasm in female dogs and pose a significant health challenge [1]. They can be classified as benign or malignant, with benign tumors having an excellent prognosis, while malignant mammary tumors show a wide variation in survival outcomes [2]. Among the malignant tumors, carcinomas are the most common and typically present a more aggressive behavior, often leading to a more challenging prognosis [2]. Surgery is considered the gold-standard treatment, as it allows for the histopathological classification of the tumor and offers the possibility of a cure in non-metastatic cases [3].

Although surgery is widely accepted as the standard treatment, there is no consensus on which surgical technique provides the best balance between the complete removal of neoplastic tissue and minimizing the risks of complications and recurrences [4,5]. Studies such as Stratmann et al. (2008) [6] report a high frequency of recurrence in remaining mammary glands after regional mastectomy, suggesting a preference for more invasive techniques, such as unilateral mastectomy. On the other hand, more invasive techniques may be associated with higher chances of postoperative complications [7,8,9] and greater impact on immune suppression [10].

These complications and recurrences can influence the survival of female dogs. However, other studies have not identified a significant association between the type of mastectomy and tumor recurrence time or survival [7,11,12]. Despite the relevance of this topic, the association between mastectomy technique and the survival of female dogs remains underexplored, highlighting the need for further studies.

Based on this, we hypothesize that more invasive mastectomy techniques, such as unilateral or bilateral mastectomy, may be associated with a better prognosis by enabling the removal of a larger amount of mammary tissue and regional lymph nodes. Conversely, less invasive techniques, such as lumpectomy, could increase the risk of recurrence by leaving residual neoplastic tissue, potentially compromising the prognosis. Thus, this study aims to evaluate the influence of the surgical procedure type used to treat mammary carcinomas on the survival of female dogs.

## 2. Materials and Methods

### 2.1. Ethical Approval

The study was approved by the Institutional Animal Care and Use Committee of the Federal University of Uberlandia under protocol 104/2022.

### 2.2. Study Design

A retrospective study was conducted, selecting intact female dogs voluntarily brought by their owners for surgical treatment of mammary carcinoma at the Veterinary Hospital of the Federal University of Uberlândia (HOVET—UFU) between January 2012 and December 2022. Each clinical record included a consent form signed by the owner, authorizing the inclusion of the animal in the study.

### 2.3. Inclusion Criteria

The inclusion criteria were as follows: female dogs clinically evaluated and deemed fit for surgical treatment; previously subjected to thoracic radiographs and abdominal ultrasonography to assess pulmonary and abdominal metastases, respectively; and undergoing surgical procedures for mammary tumor excision, with histopathological diagnosis confirming a mammary carcinoma [13].

### 2.4. Exclusion Criteria

Exclusion criteria included the following: patients with only benign neoplasms, those with malignant tumors other than carcinomas, and cases in which contact with the owner was lost during follow-up, which prevented the collection of survival data. Additionally, dogs subjected to adjuvant therapies, such as cyclooxygenase inhibitors (administered at any time from diagnosis to the postoperative period, regardless of duration), chemotherapy, or ovariohysterectomy prior to mastectomy were excluded. Dogs that died from causes unrelated to mammary carcinoma or whose survival status could not be determined were also excluded, as in Horta et al. (2014) [7].

### 2.5. Animal Grouping by Age

Based on age, the animals were grouped as puppies (<1 year), adults (1–8 years), and seniors (>8 years) [14].

### 2.6. Surgical Techniques

Given the complexity and variability of mammary tumors, the choice of mastectomy technique was individualized. Indications served as general guidelines, adjusted to meet each patient’s specific needs. The choice was based on the patient’s health status, the number, size, and location of lesions, and the presence of ulceration [4,5,15].

The techniques employed included lumpectomy, simple mastectomy, regional mastectomy, unilateral mastectomy, bilateral mastectomy, and combinations of these techniques. Unilateral mastectomy was prioritized, avoiding lumpectomy and simple mastectomy as recommended by Cassali et al. (2020) [4], to reduce recurrences and the need for additional surgeries.

Unilateral mastectomy was the first choice for female dogs with one or more tumors located in a single mammary chain, provided there was sufficient skin for synthesis and the patient could tolerate a more invasive anesthetic and surgical procedure.

Female dogs with distant tumors in both mammary chains and insufficient skin for bilateral techniques underwent two unilateral mastectomy procedures approximately 30 days apart. In these cases, chains with ulcerated or larger tumors were prioritized for the first procedure. Female dogs with tumors located at opposite extremes of both mammary chains and adequate skin availability underwent bilateral mastectomy to avoid multiple surgeries.

Regional mastectomy was performed in accordance with local lymphatic drainage [6] in female dogs with tumors located in both mammary chains or in cases of single tumors where there was insufficient skin availability to perform a complete unilateral mastectomy.

Female dogs with distant tumors in both mammary chains, adequate skin availability, and anesthetic limitations underwent bilateral mastectomy to avoid multiple surgeries.

Lumpectomy and simple mastectomy were performed on female dogs with tumors smaller than 1 cm and 3 cm, respectively, particularly in those with ulcerated lesions but with restrictions for more invasive anesthetic and surgical procedures, such as cardiac, respiratory, hepatic, or renal conditions.

A surgical margin of 1–2 cm around the tumor was maintained, as recommended by Cassali et al. (2020) [4]. Inguinal lymph nodes were removed en bloc with the inguinal mammary gland whenever this gland was excised. Similarly, axillary lymph nodes were removed en bloc when tumors were located in the cranial thoracic, caudal thoracic, or cranial abdominal mammary glands, or if the lymph nodes exhibited alterations in shape, volume, or consistency. All lymph nodes were subsequently evaluated histopathologically [13].

### 2.7. Histopathological Diagnosis

The histopathological reports were reviewed in a retrospective study. All histopathological diagnoses had been previously performed at the animal pathology laboratory of the veterinary hospital where the study was conducted. Tissue lesions from mammary tumor samples fixed in 10% buffered formalin were originally examined on hematoxylin and eosin-stained slides [16]. These diagnoses were established through a standardized double-blind evaluation by two trained pathologists, using the classification system by Goldschmidt et al. (2011) [13] as a reference. In this retrospective study, we assessed the recorded diagnoses but did not conduct new slide analyses. Mammary tumors were categorized as benign or malignant and histologically graded as I, II, and III according to the Nottingham system modified by Elston and Ellis (1991) [17]. Female dogs with malignant tumors were staged using the Tumor–Node–Metastasis (TNM) system [4]. Although variations between pathologists may occur, the histopathological analyses followed standardized methodologies routinely applied in our laboratory, as described in this study, ensuring consistency in diagnostic criteria.

### 2.8. Survival Monitoring

Survival time was defined as the period (in days) from the first surgery to death. The female dogs were clinically monitored quarterly until death or for a minimum of two years.

Telephone contact was made with the owners to collect survival data for at least one year following surgery. Recurrence or the development of new tumors in the remaining mammary glands or distant organs, the time between surgery and recurrence or new tumor development were registered. The exact date of death (day, month, and year) was recorded, or if unavailable, the approximate date (month and year) was noted. In such cases, the first day of the indicated month was considered. Cases in which contact with the owner was lost were excluded from survival analysis.

### 2.9. Statistical Analysis

Statistical analysis treated each animal as a single observation. Each surgical technique represented a group: lumpectomy, simple mastectomy, regional mastectomy, unilateral mastectomy, bilateral mastectomy, and combinations of techniques. All statistical analyses were performed using commercial software (IBM SPSS Statistics v. 19, IBM, Somers, NY, USA, and Prism v. 5.0, GraphPad, San Diego, CA, USA) [18].

Overall survival time was analyzed using the Kaplan–Meier method, with the log-rank test employed to compare survival curves according to surgical technique. Cox regression univariate analysis was used to compare mastectomy types and overall survival time. Cases were censored for analysis when clinical follow-up was lost. To evaluate the relationship between histological subtype and the type of mastectomy performed, as well as between the type of mastectomy and histological grade, and the association between the type of mastectomy and the recurrence of new tumors in the postoperative period, the Chi-square test was used for categorical variables. The correlation between clinical stage and surgical technique, as well as between stage and age, was analyzed using Spearman’s rank correlation test. Differences in age among groups for each surgical technique were assessed using ANOVA. For each analysis, only female dogs with complete data for the variables evaluated were included. The statistical level of significance for all tests was set at 5% (*p* < 0.05).

The analyses conducted to evaluate the association between clinical stage and surgical technique, as well as between stage and age, histological subtype and surgical technique, and histological grade and surgical technique, were performed to ensure that well-established prognostic factors, such as age, stage, histological subtype, and grade, were equally distributed among the groups. This approach aimed to avoid potential biases in the survival analysis caused by an unequal distribution of these factors across different surgical techniques. By confirming that the groups were comparable in terms of prognostic variables, we sought to strengthen the validity of the survival analysis, which was the primary objective of the study. These factors were included as prognostic markers, not as determinants of the surgical decision.

## 3. Results

Out of 724 female dogs with mammary neoplasms that underwent mastectomy, 307 met the inclusion criteria for the study, having at least one mammary carcinoma diagnosed histopathologically, which could be accompanied by other benign mammary tumors, but without other malignant histological subtypes. This comprised a total of 734 mammary tumors. The dogs’ ages ranged from 1 to 17 years (mean 9.4 ± 3.1 years).

There were 150 (48.9%) mixed-breed dogs and 3 (1%) with no breed reported, while the remaining 154 dogs represented 22 different breeds. Poodles were the most affected breed (*n* = 46; 15%), followed by Shih Tzus (*n* = 21; 6.8%) and Pinschers (*n* = 20; 6.5%) (Table 1).

One hundred and ninety (62%) of the 307 dogs had more than one mammary lesion, and 30% had tumors in both mammary chains. Histopathological diagnoses were established for 734 tumors, with 682 (92.9%) classified as malignant neoplasms and 52 (7.1%) as benign neoplasms.

Benign mixed tumors represented 28.9% (15/52) of the benign neoplasms, followed by simple adenomas (25%, 13/52) and complex adenomas (19.2%, 10/52) (Table 2). Carcinoma in mixed tumors was the most frequent malignant neoplasm (30.2%), followed by complex carcinomas (19.1%) and tubular carcinomas (17.2%).

Tumor size was reported in 646 cases, with 428 (66.3%) being smaller than 3 cm (T1), 136 (21.1%) measuring between 3 and 5 cm (T2), and only 82 (12.7%) exceeding 5 cm (T3). Histological grading revealed that 51.5% of the tumors were grade I (284/551), 41.6% were grade II (229/551), and 6.9% were grade III (38/551). Regarding TNM staging, 33.0% of the dogs were in stage I (62/188), 15.4% in stage II (29/188), 11.2% in stage III (21/188), 22.9% in stage IV (43/188), and 17.6% in stage V (33/188).

Regarding the type of mastectomy performed for surgical treatment of mammary carcinomas, of the 307 dogs, 3 (1%) underwent lumpectomy, 22 (7.2%) simple mastectomy, 12 (3.9%) regional mastectomy, 202 (65.8%) unilateral mastectomy, 7 (2.3%) bilateral mastectomy, and 61 (19.9%) combined procedures involving both mammary chains. Of these 61 dogs, 41 (67.2%) underwent two-staged unilateral mastectomies at intervals, 12 (19.7%) unilateral mastectomy and regional mastectomy, 6 (9.8%) unilateral mastectomy and simple mastectomy, 1 (1.6%) regional and simple mastectomy, and 1 (1.6%) regional mastectomy and lumpectomy (Table 3).

The relationship between histological type and the type of mastectomy performed was assessed, with lumpectomy excluded from the analysis due to its low frequency. Only the most common carcinomas (mixed tumor carcinoma, complex carcinoma, tubular carcinoma, tubulopapillary carcinoma, and solid carcinoma) were considered. The Chi-square test showed no association between the type of mastectomy and the type of mammary carcinoma (*p* = 0.2), nor between the type of mastectomy and histological grade (*p* = 0.09). Spearman’s rank correlation test revealed no correlation between the patient’s stage and the surgical technique (*p* = 0.1). There was also no difference in age between the groups for each type of mastectomy (*p* = 0.6).

During the postoperative period, 48 (15.6%) dogs developed new tumors in the remaining mammary tissue (Table 4), with the mean time for new lesions ranging from 5.5 to 84 months. New lesions were observed in 66.7% (2/3) of dogs undergoing lumpectomy, 18.2% (4/22) of those undergoing simple mastectomy, 16.7% (2/12) undergoing regional mastectomy, 16.3% (33/202) undergoing unilateral mastectomy, 14.3% (1/7) undergoing bilateral mastectomy, and 9.8% (6/61) undergoing combined techniques (*p* = 0.2). Of the 48 cases, 43.8% (21/48) were confirmed through histopathological examination, 6.3% (3/48) were confirmed via cytological analysis, 16.7% (8/48) were identified by the owner and subsequently evaluated by the veterinarian, but without cytological or histopathological confirmation, and 33.3% (16/48) were assessed by palpation performed by the owner.

The average follow-up period was 1030 days (range, 1–4251), during which 181 animals died. The mean overall survival for dogs with mammary carcinoma was 1593 days (95% confidence interval [CI], 1398–1787), and the median overall survival was 1346 days (95% CI, 1000–1691). Of these 307 animals, 126 (41.0%) were censored for being alive until the date of the last follow-up, with none having undergone lumpectomy, 4 (3.2%) undergoing simple mastectomy, 5 (4%) regional mastectomy, 81 (64.3%) unilateral mastectomy, 2 (1.6%) bilateral mastectomy, and 34 (27%) a combination of techniques.

In assessing the correlation between survival and mastectomy type, the highest mortality rate was observed in dogs treated with lumpectomy (3/3—100%), followed by simple mastectomy (20/22—90.9%), bilateral mastectomy (6/7—85.7%), regional mastectomy (9/12—75%), combined techniques (35/61—57.4%), and unilateral mastectomy (108/202—53.5%).

The mean survival for dogs undergoing lumpectomy was 179 days, with a median of 103 days; for simple mastectomy, the mean was 1343 days, and the median was 1651 days; for regional mastectomy, the mean was 1654 days, and the median was 1651 days; for unilateral mastectomy, the mean was 1639 days, and the median was 1654 days; for bilateral mastectomy, the mean was 1464 days, and the median was 1057 days; and for combined techniques, the mean was 999 days, and the median was 957 days.

**Figure 1:**
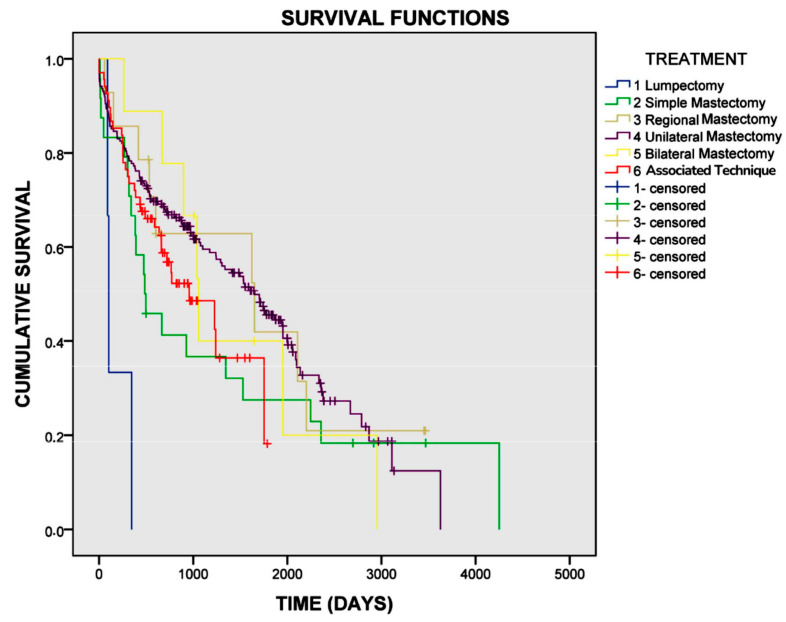
Survival curve for female dogs with mammary carcinomas based on the type of mastectomy performed. The mean overall survival for dogs undergoing lumpectomy was 179 days, for simple mastectomy was 1343 days, for regional mastectomy was 1654 days, for unilateral mastectomy was 1639 days, for bilateral mastectomy was 1464 days, and for the combination of techniques was 999 days (*p* = 0.006).

The log-rank test indicated a correlation between survival and the type of mastectomy performed (*p* = 0.006) (Figure 1). In the univariate Cox regression analysis, dogs undergoing lumpectomy were 4.9 times more likely to die compared to those treated with other types of mastectomy (*p* = 0.009).

## 4. Discussion

Mammary carcinomas were more frequent in older female dogs, with a mean age of 9.4 years, in agreement with the literature [6,7,19]. This finding reinforces the need for more rigorous monitoring of older dogs for the early detection of neoplasms. The predominance of mixed-breed dogs in this study can be attributed to the profile of the population served by a public hospital. This pattern was also observed by Daleck et al. (1998) [19], who reported a higher frequency of mixed-breed dogs compared to purebred dogs, especially in environments with greater access to public veterinary services. The study revealed that the Poodle breed was the most affected among the dogs with mammary neoplasms, consistent with studies showing a higher risk of developing mammary tumors in dogs of this breed [20,21].

Although dogs with only benign tumors were censored from the study, the frequency of benign mammary tumors coexisting with malignant ones was low, consistent with what is reported in the literature. De Nardi et al. (2002) [22] cited a 56% occurrence of malignant tumors in dogs, followed by Sorenmo (2003) [23] with 50%, Stratmann et al. (2008) [6] with 68.4%, Oliveira Filho et al. (2010) [24] with 73.3%, and Horta et al. (2014) [7] with 74%.

Variations in these percentages may be related to regional characteristics, such as the use of contraceptives [22] and delays in seeking veterinary care [7]. Horta et al. (2014) [7], as well as this study, also reported that carcinoma in mixed tumors was the most frequent malignant tumor, representing 47.5% of the lesions, followed by carcinoma in situ (23.3%). On the other hand, Oliveira Filho et al. (2010) [24] reported simple carcinoma as the most frequent, representing 40% of all malignant neoplasms. Malignant tumors differ in their aggressiveness according to their histological classification, with simple carcinomas being more aggressive than complex carcinomas and carcinomas in mixed tumors [2].

Unilateral mastectomy was the most commonly performed technique in this study (65.8%), followed by the association of procedures involving both mammary chains (19.9%), as reported by Horta et al. (2014) [7], Hörnfeld and Mortensen (2023) [25], and Soares et al. (2023) [26]. In contrast, bilateral mastectomy, a more invasive technique, and lumpectomy, a less invasive technique, were rarely employed. Stratmann et al. (2008) [6] observed that 57.6% of dogs with mammary carcinoma treated with regional mastectomy developed new tumors in the ipsilateral mammary tissue, with 77% being malignant. These authors recommended total unilateral mastectomy to avoid further surgical interventions. On the other hand, Horta et al. (2014) [7] found no association between the mastectomy technique and recurrence or survival, recommending the removal of mammary tumors by the simplest procedure that ensures complete removal of the lesion and the main lymphatic connections.

The high frequency of dogs that developed new tumors in the remaining mammary tissue in this study underscores the importance of postoperative monitoring for early detection of new lesions and the implementation of appropriate therapeutic measures. Lumpectomy was the technique that showed the highest frequency of new lesions and the shortest interval for their development. However, no significant association was found between the surgical technique and the development of new tumors, likely due to the low number of dogs undergoing lumpectomy and bilateral mastectomy. Still, we believe that the lack of adequate surgical margins, the possibility of residual neoplastic tissue, and the presence of regional lymph nodes may facilitate the spread of tumor cells after surgery, compromising the prognosis [4,27]. Lumpectomy was associated with a shorter survival time. While other studies have recommended lumpectomy for small, non-invasive, single tumors [5,28,29], this finding supports the recommendation of Cassali et al. (2020) [4] that this technique should be avoided.

Unlike previous studies, which did not identify an association between surgical technique and overall survival or disease-free interval [7,11,26,30], this was the first study to demonstrate a significant reduction in survival for dogs treated with lumpectomy. However, there was no significant difference in survival between the other techniques evaluated, consistent with previous studies. Thus, the choice of surgical technique should consider other factors, such as the dog’s clinical condition, the number of mammary lesions, and the achievement of adequate surgical margins. Clean margins are a relevant prognostic factor, associated with better outcomes and lower recurrence rates [2]. In mammary tumors, a safety margin of at least 1–2 cm of healthy tissue is recommended, which may involve adjacent musculature in the deep plane (such as the pectoral and oblique or rectus abdominal muscles) in cases of tumor adherence [4]. This standard cannot be achieved with the lumpectomy technique. Based on the results obtained, lumpectomy is recommended only for specific cases, such as dogs with clinical restrictions for more invasive procedures and with ulcerated and painful tumors.

In human medicine, lumpectomy is more commonly used in the treatment of early-stage breast cancer, often in combination with adjuvant therapies such as radiotherapy. Clinical trials have shown that breast-conserving therapy, including lumpectomy with adjuvant radiotherapy, can be effective and safe for women with early-stage breast cancer [31,32]. These studies have also shown no significant difference in survival rates between women undergoing breast-conserving mastectomy with radiotherapy and those undergoing radical mastectomy [33]. The study by Boughey et al. (2023) [32] reported a local recurrence rate of 3.1% over 5 years in women who underwent breast-conserving therapy with negative margins and radiation boost to lumpectomy beds. This low recurrence rate highlights the importance of combining conservative surgery and radiotherapy for oncological control in humans.

In contrast, adjuvant radiotherapy remains underexplored in dogs, primarily due to limitations in availability and associated costs, which represents a significant gap in the oncological management of mammary neoplasms in animals. Additionally, while the choice of conservative surgery in human medicine is often linked to the preservation of breast aesthetics, this aesthetic concern is not as relevant in dogs, where the primary focus is on oncological control and increasing survival time.

The absence of an association between key prognostic factors—such as age, clinical stage, histological type, and histological grade of mammary carcinoma—and the surgical techniques employed suggests that these variables did not affect the survival outcomes based on the surgical approach. This finding reinforces the notion that these prognostic factors were equally distributed across the groups, ensuring that the comparison of surgical techniques was unbiased. Moreover, since factors like histological grade and subtype are typically determined postoperatively, their influence on the surgical decision-making process is limited, further supporting the argument that these factors did not influence group assignment. Therefore, the results more accurately reflect the impact of the surgical techniques on survival. However, the small sample size in some groups should be taken into account, as it may limit the generalizability and robustness of these findings.

The limitations of this study include reliance on owner-reported data, which may be subject to recall bias or inaccuracies, and the unconfirmed occurrence of new tumors in some cases, which relied on the owner’s report. Excluding cases due to loss of follow-up or deaths unrelated to the tumor was necessary to maintain the validity of the survival analysis but may limit generalizability. Missing data, such as histological grade and tumor size, were due to the retrospective nature of the study. The small number of cases in the lumpectomy group limits the robustness of the analysis, and the absence of data on histologic tumor-free margins, which were not systematically evaluated, is another important limitation.

## 5. Conclusions

Based on the available data, lumpectomy appears to be associated with shorter survival times in female dogs with mammary carcinoma. Therefore, it may be advisable to reconsider this technique as a primary treatment option. More invasive approaches, such as unilateral mastectomy, should be prioritized where feasible, although the small sample size in certain groups calls for caution in drawing definitive conclusions.

## Figures and Tables

**Table 1:** Breeds and age of female dogs with mammary carcinoma undergoing mastectomy from 2012 to 2022 at the Veterinary Hospital of the Federal University of Uberlândia, Minas Gerais, Brazil.

Breed	Frequency (%)
Mixed breed	150 (48.9%)
Poodle	46 (15%)
Shih Tzu	21 (6.8%)
Pinscher	20 (6.5%)
Yorkshire	10 (3.3%)
Basset Hound	9 (2.9%)
Dachshund	7 (2.3%)
Maltese	7 (2.3%)
Pitbull	5 (1.6%)
Lhasa Apso	5 (1.6%)
Boxer	4 (1.3%)
Labrador	3 (1%)
Cocker Spaniel	3 (1%)
Blue Heeler	2 (0.7%)
Beagle	2 (0.7%)
Dalmatian	2 (0.7%)
Schnauzer	2 (0.7%)
Fila	1 (0.3%)
Border Collie	1 (0.3%)
German Shepherd	1 (0.3%)
Swiss Shepherd	1 (0.3%)
Rottweiler	1 (0.3%)
Fox Terrier	1 (0.3%)
Not reported	3 (1%)
Total	307
Age	
Puppy	1 (0.3%)
Adult	126 (41.1%)
Senior	176 (57.3%)
Not reported	4 (1.3%)
Total	307

**Table 2:** Frequency of carcinomas and benign mammary tumors in 734 mammary tumors of 307 bitches included in the survival analysis, according to mastectomy technique, from 2012 to 2022, at the Veterinary Hospital of the Federal University of Uberlândia, Minas Gerais, Brazil.

Histological Subtype	Frequency (%)
Carcinomas	
Mixed Tumor Carcinoma	206 (30.2%)
Complex Carcinoma	130 (19.1%)
Tubular Carcinoma	117 (17.2%)
Tubulopapillary Carcinoma	85 (12.5%)
Solid Carcinoma	63 (9.2%)
Carcinoma in situ	28 (4.1%)
Papillary Carcinoma	21 (3.1%)
Micropapillary Carcinoma	12 (1.8%)
Anaplastic Carcinoma	11 (1.6%)
Ductal Carcinoma	9 (1.3%)
Total	682
Benigns	
Benign Mixed Tumor	15 (28.9%)
Simple Adenoma	13 (25%)
Complex Adenoma	10 (19.2%)
Adenomyoepithelioma	7 (13.5%)
Myoepithelioma	4 (7.7%)
Tubular Adenoma	2 (3.9%)
Fibroadenoma	1 (1.9%)
Total	52

**Table 3:** Types of mastectomy used for the surgical treatment of female dogs with mammary carcinoma, from 2012 to 2022, at the Veterinary Hospital of the Federal University of Uberlândia, Minas Gerais, Brazil.

Type of Mastectomy	Frequency (%)
Unilateral mastectomy	202 (65.8%)
Simple mastectomy	22 (7.2%)
Regional mastectomy	12 (3.9%)
Bilateral mastectomy	7 (2.3%)
Lumpectomy	3 (1%)
Combination of procedures	61 (19.9%)
Total	307
Combination of procedures	
Unilateral mastectomy and unilateral mastectomy	41 (67.2%)
Unilateral mastectomy and regional mastectomy	12 (19.7%)
Unilateral mastectomy and simple mastectomy	6 (9.8%)
Regional mastectomy and simple mastectomy	1 (1.6%)
Regional mastectomy and lumpectomy	1 (1.6%)
Total	61

**Table 4:** Number of bitches that developed new mammary tumors after mastectomy for the treatment of mammary carcinomas, according to the surgical technique and the average time for recurrence development after surgical treatment, from 2012 to 2022, at the Veterinary Hospital of the Federal University of Uberlândia, Minas Gerais, Brazil.

Surgical Technique	New Lesions in Remaining Mammary Gland	$\bar{x}$ (Months)
Lumpectomy	66.7% (2/3)	5.5
Simple mastectomy	18.2% (4/22)	13.8
Regional mastectomy	16.7% (2/12)	12.3
Unilateral mastectomy	16.3% (33/202)	12.5
Bilateral mastectomy	14.3% (1/7)	84
Association of techniques	9.8% (6/61)	12.2
Total	48	

## Data Availability

All the available data are included in the manuscript. Additional information can be obtained from the corresponding author.
